# G-CSF promotes the viability and angiogenesis of injured liver via direct effects on the liver cells

**DOI:** 10.1007/s11033-022-07715-4

**Published:** 2022-07-04

**Authors:** Zifeng Liu, Guiling Zhang, Jing Chen, Jingjing Tong, Hongmin Wang, Jing Chen, Dong Yang, Jinhua Hu

**Affiliations:** 1 Medical School of Chinese PLA, Beijing, China; 2 Senior Department of Hepatology, The Fifth Medical Center of PLA General Hospital, Beijing, China; 3 Department of Pathology, Chengwu People’s Hospital, Heze, China; 4 Department of Infectious Diseases, Beijing Jishuitan, Beijing, China; 5 Peking University 302 Clinical Medical School, Beijing, China; 6 Oncology Department, Affiliated Hospital of Jining Medical University, Jining, China

**Keywords:** Liver failure, Granulocyte-colony stimulating factor, Vessel endothelial growth factor A, Cell viability, Angiogenesis

## Abstract

**Background:**

Presently, liver transplantation is the only treatment strategy for liver failure (LF). Although granulocyte-colony stimulating factor (G-CSF) exhibits protective functions in LF, it is not clear whether it directly affects the liver cells.

**Methods and Results:**

We established an injured liver cell model and observed that G-CSF treatment promoted cell viability and enhanced Ki67 and VEGF-A expression. Thereafter, human umbilical vein endothelial cells (HUVECs) were cultured in a conditioned medium collected from the G-CSF-treated injured liver cells. HUVECs’ proliferation and tubule formation were promoted. Furthermore, in an injured liver mouse model, confirmed via haematoxylin–eosin staining, we evaluated serum alanine aminotransferase activity, Ki67 expression, and microvessel density (MVD). G-CSF treatment significantly relieved liver injury, upregulated Ki67 expression, and enhanced MVD in the injured mouse liver tissue. Additionally, AKT and ERK signal targets were explored, and it was demonstrated that the effects of G-CSF on injured liver cells were mediated through the AKT and ERK signalling pathways.

**Conclusions:**

G-CSF promotes injured liver viability and angiogenesis by directly affecting injured liver cells via the AKT and ERK signalling pathways. These findings improve our understanding of the role of G-CSF in recovery from LF.

**Supplementary Information:**

The online version contains supplementary material available at 10.1007/s11033-022-07715-4.

## Introduction

Liver failure (LF) is a serious clinical condition that can result in multi-organ failure, and eventually, death [1]. Among hospitalized patients with liver insufficiency and alcoholic cirrhosis, the occurrence of LF is as high as 28%. Presently, liver transplantation is considered the most effective treatment approach for LF [2]; however, it is inaccessible to most patients owing to the high cost and shortage of donor organs [3, 4]. Thus, it is necessary and urgent for clinicians to find an effective treatment for LF.

Granulocyte-colony stimulating factor (G-CSF), a growth factor secreted by various types of cells, including macrophages, endothelial cells, and some immune cells, can induce growth and differentiation of neutrophils and neutrophilic progenitor cells in the bone marrow (BM) [5, 6]. It has been used in patients for chemotherapy-induced BM suppression and severe chronic neutropenia resulting from various causes [7, 8]. Recently, the protective function of G-CSF in LF has attracted considerable attention. In rats, G-CSF accelerated liver regeneration by inducing the migration of BM-derived progenitors to the liver and by increasing endogenous oval cell response [9]. Furthermore, in a clinical trial, G-CSF therapy promoted the mobilization of CD34( +) cells in patients with hepatitis B virus-associated acute-on-chronic liver failure (ACLF) and improved liver function [10]. Although the protective role of G-CSF in LF can be attributed to the induction of BM hematopoietic stem cells (HSCs) to differentiate into liver cells [11, 12], whether G-CSF directly affects liver cells is yet to be elucidated.

In this study, we explored the direct roles of G-CSF in viability and VEGF-A expression in injured liver cells, and the indirect stimulation on human umbilical vein endothelial cells (HUVECs).In addition, we investigated the role of the AKT and ERK signalling pathways. Our findings will enable better understanding of the mechanism underlying the role of G-CSF in potential treatments for LF.

## Materials and methods

### Cell culture

Human foetal liver cell line (LO2) and human hepatoma cell line (HepG2), widely used for establishing injured liver cell models [13, 14], were obtained from the Senior Department of Infectious Diseases, the Fifth Medical Center of PLA (People’s Liberation Army) General Hospital. Human umbilical vein endothelial cells (HUVECs) were obtained from Otwo Biotech (Guangzhou, China). LO2 and HepG2 cells were cultured in Dulbecco’s modified Eagle’s medium (DMEM; Invitrogen, Shanghai, China), containing 10% foetal bovine serum (FBS; Gibco Life Technologies, Grand Island, NY, USA), under 5% CO_2_ at 37 °C, while the HUVECs were cultured in endothelial cell medium (ECM; Sciencell, Carlsbad, CA, USA), containing 5% FBS and 1% endothelial cell growth factor (ECGS; Sciencell) in a 5% CO_2_ atmosphere at 37 °C.

### Establishment of the injured liver cell model

LO2 and HepG2 cells (1.5 × 10^5^ cells/well) were seeded in 96-well plates. After 24 h, D-galactosamine (D-GalN; ST1213, Beyotime Biotechnology, Shanghai, China) was added at final concentrations of 6, 8, 10, and 12 mg/mL, and CCK8 assays (K1018, APExBIO Technology LLC, Houston, TX, USA) were performed after 24 h to confirm cell injury. Thereafter, appropriate D-GalN concentration and treatment duration were adopted for the subsequent experiments.

### Collection of conditioned medium (CM)

LO2 and HepG2 cells (2 × 10^5^ cells/well) were seeded in 6-well plates and cultured in the presence of D-GalN (final concentration, 10 mg/mL) for 24 h. The experimental group was treated with G-CSF at a final concentration of 10,000 ng/mL; the total culture time (72 h) included 24 h before, 24 h during, and 24 h after D-GalN treatment. Thereafter, the medium was replaced with ECM and the cells were further cultured for 48 h. Finally, the supernatant was collected as the CM for subsequent experiments.

### CCK8 assay

The CCK8 assay (K1018, APExBIO Technology LLC) was performed following the manufacturer’s protocol. For injured liver cells and their counterparts treated with varying concentrations of G-CSF (500, 1,000 and 10,000 ng/mL), CCK8 assays were performed at 0, 24, 48, 72, and 96 h.

HUVECs were plated and incubated for 24 h, and the culture medium was replaced with CM from untreated injured liver cells and G-CSF-treated injured liver cells (10,000 ng/mL). The CCK8 assay was performed 48 h later.

### Quantitative real time-polymerase chain reaction (qRT-PCR)

RNA was extracted from the cells using TRIzol reagent (15596018, Invitrogen, Carlsbad, CA, USA), according to the manufacturer’s instructions. Thereafter, cDNA was synthesized using the PrimeScript RT-PCR Kit (FSQ-301, Toyobo, Osaka, Japan). Next, qRT-PCR was performed using the cDNA as a template and the Universal PCR Master Mix (CW0957M, Beijing Cowin Biotech Co., Ltd., Beijing, China) on a CFX96 Touch Deep Well™ Real-Time PCR Detection System (Bio-Rad, Hercules, CA, USA). The amplification results were then quantitated using the 2^(−ΔΔCt)^ method. Sequences of the primers used are shown in Supplement Table 1.

### Western blotting

Total protein was separated and transferred onto polyvinylidene difluoride membranes (Millipore Corp., Billerica, MA, USA). Thereafter, the membranes were incubated overnight with primary antibodies (Supplement Table 2) at 4 °C and in peroxidase-linked goat anti-rabbit-IgG (1:5,000; ab6721, Abcam, Cambridge, UK) and goat anti-mouse-IgG (1:5,000; ab6789, Abcam) at room temperature (approximately 25 °C) for 1 h. The blots obtained were developed using enhanced chemiluminescence reagents (32106, Pierce, Rockford, IL, USA), and analyzed quantitatively using ImageJ software (National Institute of Health, Bethesda, MD, USA).

### Tubule formation assay

HUVECs (3 × 10^4^ cells/well) were plated in Matrigel (356234, Corning Inc., Corning, NY, USA)-precoated 48-well plates. After culturing in CM for 12 h, the tubular structures were quantified via manual counting at three random × 100 fields. Thereafter, the number of structures was averaged.

### Establishment of the injured liver mouse model

A total of 18 male C57BL/6 mice (Pengyue Laboratory Animal Breeding Co., Ltd., Jinan, China) were assigned to the control (n = 4), injured liver (n = 7), and treatment (n = 7) groups. Mice in the control group were not administered any treatment. Those in the injured liver group received a peritoneal injection of D-GalN (1,000 mg/kg), whereas those in the treatment group received a subcutaneous injection of G-CSF (250 µg/kg) for 5 days before the peritoneal injection of D-GalN (1000 mg/kg). The mice were decapitated 24 h after D-GalN injection. Blood and liver samples were collected, and the latter was soaked in formalin. Haematoxylin–eosin (HE) staining of the liver tissue was performed and alanine aminotransferase (ALT) levels in the serum were detected to confirm liver injury. The animal experiments were performed in accordance with the principles of the 1975 Declaration of Helsinki and were approved by the Ethics Committee of the Fifth Medical Center of PLA General Hospital (Approval ID: IACUC-2013-022).

### ALT assay

Blood samples from the mice were centrifuged at 1000 rpm for 10 min and serum samples were collected for the ALT assay, which was performed using an ALT assay kit (C009-2-1, Nanjing Jiancheng Bioengineering Institute, Nanjing, China) in accordance with the manufacturer’s instructions.

### Immunohistochemistry (IHC)

Mouse liver samples were embedded in paraffin, cut into 3-μm-thick slices, and incubated overnight with primary antibodies against Ki67 (1:100, AF0198, Affinity Bioscience, Jiangsu, China) or CD31 (1:1500, ab182981, Abcam) at 4 °C. Normal rabbit IgG was used as the negative control. The slices were then incubated with HRP goat anti-rabbit IgG polymer (KIT-5005, Maixin, Biotechnology Development Co., Ltd., Fuzhou, China) and stained with 3,3′-diaminobenzidine, whereas the cell nuclei were stained with haematoxylin. The staining scores were simultaneously evaluated by two independent pathologists at × 200 magnification. The proportion score was recorded in at least four random fields and presented as the fraction of stained cells (0 < 10%; 1 = 10%–25%; 2 = 26%–75%; 3 > 75%). Furthermore, the intensity score represented the average staining intensity (0 = none; 1 = weak; 2 = intermediate; 3 = strong). The expression of Ki67 was determined as the product of the proportion and intensity scores. Microvessel density (MVD) was determined using CD31 immunoreactivity and quantified via manual counting at five random × 200 fields to obtain the average. Images were then scanned using the iViewer (Suzhou Youna Medical Equipment Co., Ltd., Jiangsu, China) and CaseViewer systems (3DHISTECH Ltd., Budapest, Hungary).

### Statistical analysis

All statistical analyzes were performed using SPSS software version 19.0 (IBM, Chicago, IL, USA); the data are presented as mean ± SD. Student’s *t*-test was performed to analyze the means between two groups. One-way ANOVA was performed to analyze the means among three or more groups. *p* < 0.05 was considered statistically significant. GraphPad Prism 6.0 (GraphPad Software, San Diego, CA, USA) was used to generate histograms. The experiments were performed in triplicate.

## Results

### G-CSF and granulocyte colony-stimulating factor receptor (G-CSFR) levels increased in injured liver cells

**Fig. 1:**
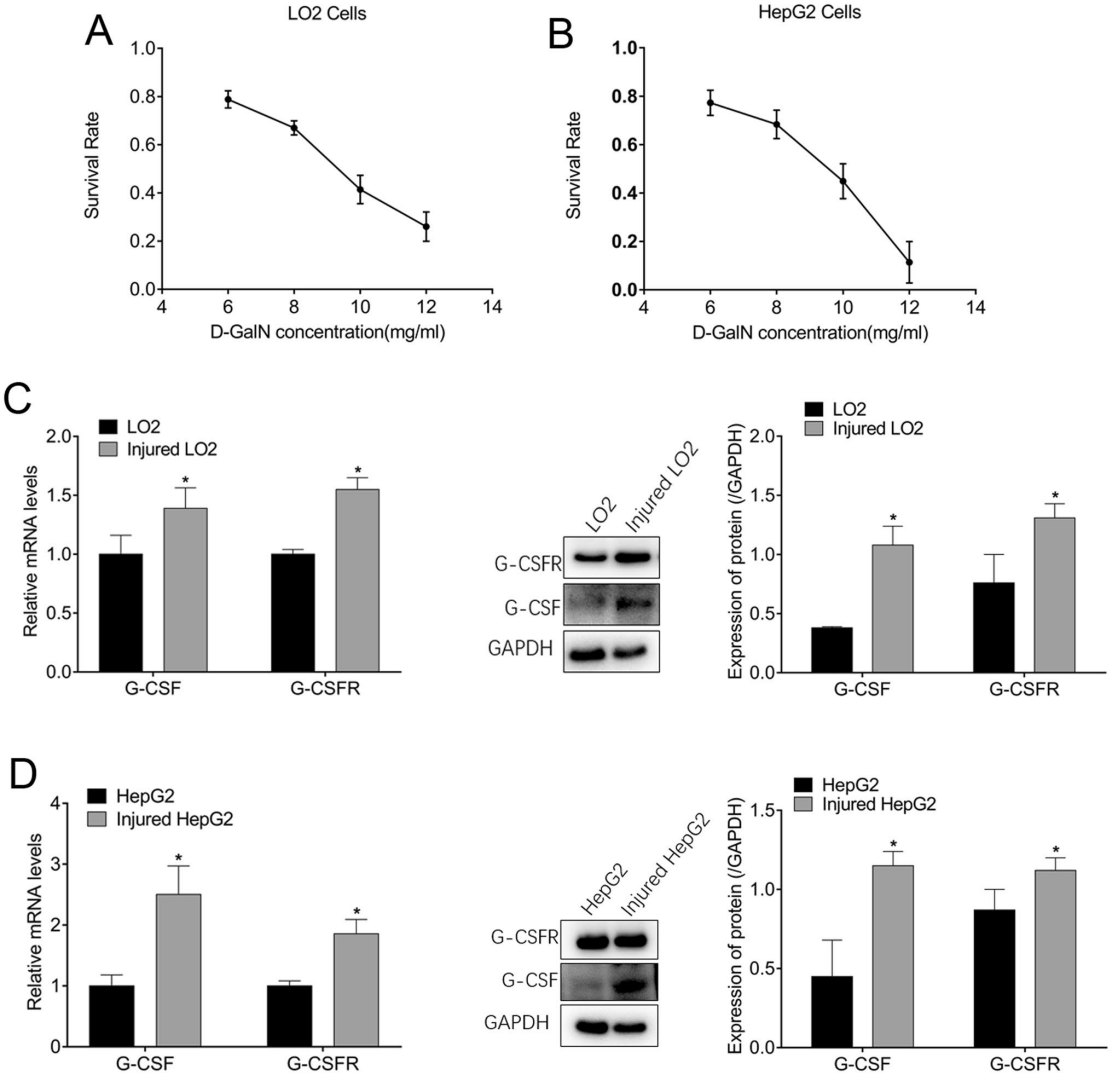
Increased levels of granulocyte-colony stimulating factor (G-CSF) and granulocyte-colony stimulating factor receptor (G-CSFR) in injured liver cells. Survival rates of (**A**) LO2 and (**B**) HepG2 cells treated with different concentrations of D-galactosamine. Expression of G-CSF and G-CSFR in LO2 and injured LO2 cells at the (**C**) RNA and protein levels. Expression of G-CSF and G-CSFR in HepG2 and injured HepG2 cells at the (**D**) RNA and protein levels. *p < 0.05

The survival rate of LO2 and HepG2 cells, 24 h after treatment with 10 mg/mL D-GalN, was 41.45% (Fig. 1A) and 44.9% (Fig. 1B), respectively, indicating the successful establishment of the injured liver cell model. Therefore, 10 mg/mL D-GalN treatment for 24 h was used in the subsequent experiments. Compared with uninjured LO2 cells, injured LO2 cells exhibited an increase in the expression of G-CSF and G-CSFR at both the RNA and protein (Fig. 1C) levels. Similar results were obtained for HepG2 cells (Fig. 1D).

### G-CSF directly promoted cell viability and VEGF-A expression in injured liver cells

**Fig. 2:**
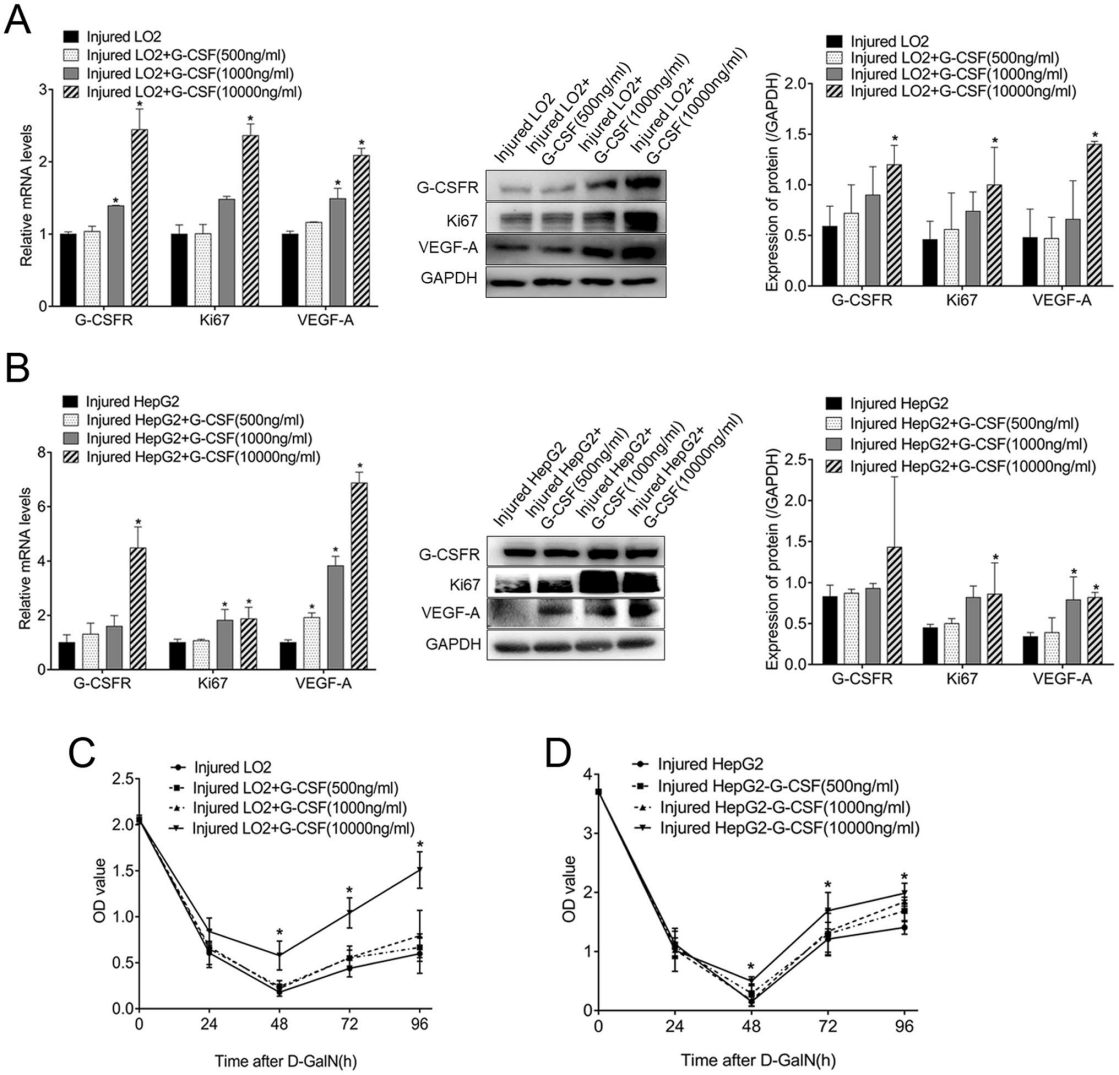
Direct promotion of injured liver cell viability and VEGF-A expression by granulocyte-colony stimulating factor (G-CSF). **A** Changes in the expression of G-CSFR, Ki67, and VEGF-A at the RNA and protein levels in injured LO2 cells treated with different concentrations of G-CSF. **B** Changes in the expression of G-CSFR, Ki67, and VEGF-A at the RNA and protein levels in injured HepG2 cells treated with different concentrations of G-CSF. **C** Changes in the viability of injured LO2 cells treated with G-CSF. **D** Changes in the viability of injured HepG2 cells after treatment with G-CSF. *p < 0.05

Compared with injured untreated LO2 cells, cells pre-treated with G-CSF at different concentrations showed higher expression of G-CSFR, Ki67, and VEGF-A both at the RNA and protein (Fig. 2A) levels; particularly, the difference was statistically significant at 10,000 ng/mL G-CSF. In HepG2 cells, similar results were obtained for the expression of G-CSFR, Ki67, and VEGF-A at the RNA level (Fig. 2B). At the protein level, the expression of Ki67 and VEGF-A showed a similar trend as that in injured LO2 cells. The expression of G-CSFR also increased after treatment with G-CSF, but the increase was not statistically significant (Fig. 2B). Compared with injured untreated LO2 cells, cell viability increased gradually in the group pre-treated with different concentrations of G-CSF, and the increase was statistically significant at 10,000 ng/mL G-CSF (Fig. 2C). Similar results were obtained in HepG2 cells (Fig. 2D).

### CM from injured liver cells treated with G-CSF stimulated HUVECs

**Fig. 3:**
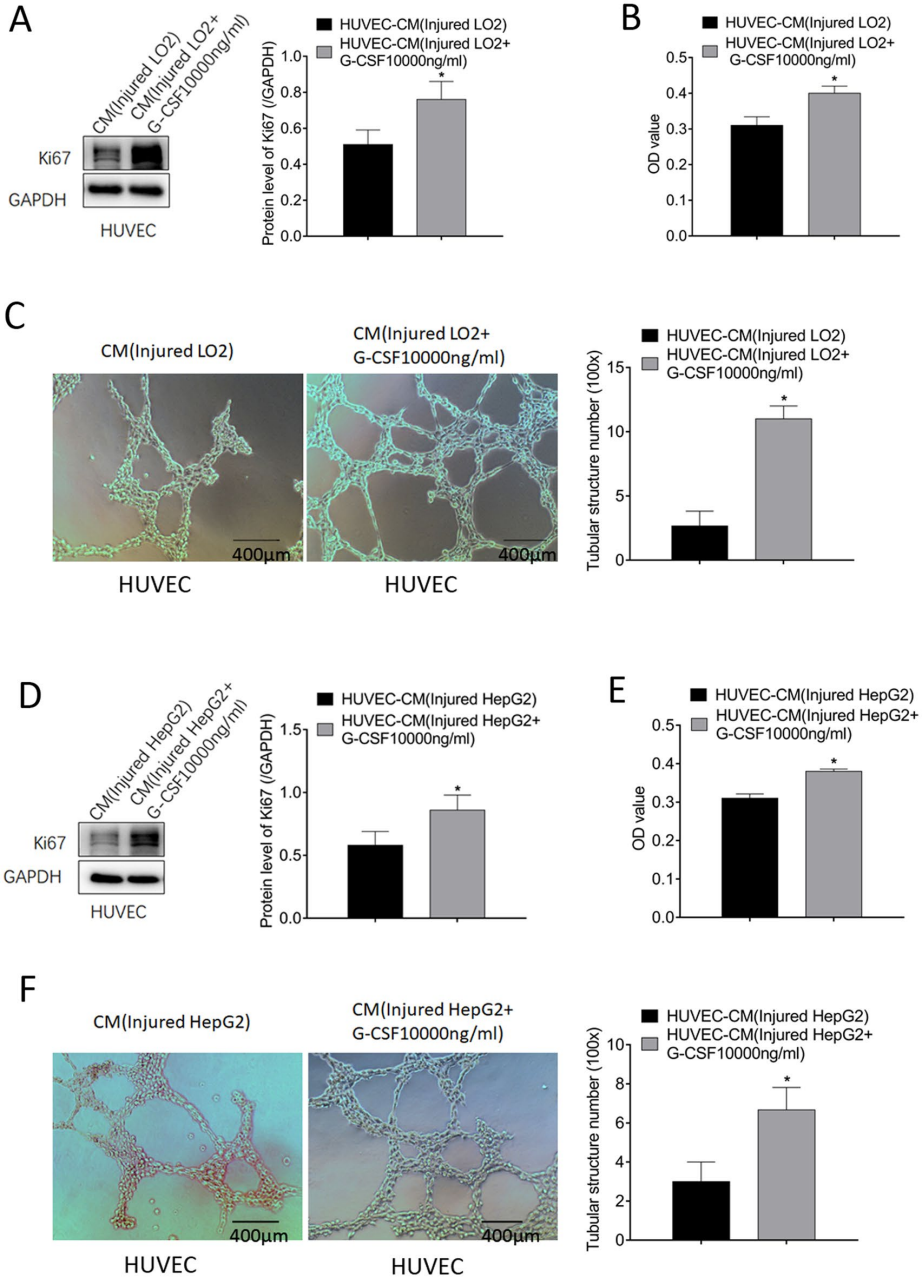
Stimulation of HUVECs by conditioned medium (CM) from injured liver cells treated with granulocyte-colony stimulating factor (G-CSF). **A** Comparison of the expression of Ki67 in HUVECs cultured with CM from injured LO2 and injured LO2 + G-CSF (10,000 ng/mL) cells. **B** Comparison of the viability of HUVECs cultured with CM from injured LO2 cells and injured LO2 + G-CSF (10,000 ng/mL) cells. **C** Comparison of tubule formation ability (× 100) following different G-CSF treatments. **D** Comparison of the expression of Ki67 in HUVECs cultured with CM from injured HepG2 and injured HepG2 + G-CSF (10,000 ng/mL) cells. **E** Comparison of the viability of HUVECs. **F** Comparison of tubule formation ability (× 100) following different G-CSF treatments. *p < 0.05

The role of G-CSF in promoting angiogenesis was further confirmed using HUVECs. Compared with HUVECs cultured in CM from injured untreated LO2 cells, those cultured in CM from injured LO2 cells treated with G-CSF exhibited a high expression of Ki67 at the protein level (*p* = 0.031) (Fig. 3A), a significant increase in cell viability (*p* = 0.001) (Fig. 3B), and an increase in the number of tubular structures (from 2.67 ± 1.15 to 11 ± 1; *p* = 0.001) (Fig. 3C). Additionally, HUVECs cultured in the CM from injured HepG2 cells treated with G-CSF showed a higher expression of Ki67 at the protein level (*p* = 0.04) (Fig. 3D), a significant increase in cell viability (*p* = 0.0002) (Fig. 3E), and an increase in the number of tubular structures (from 3 ± 1 to 6.67 ± 1.15; *p* = 0.014) (Fig. 3F).

### G-CSF relieved liver injury and showed positive correlation with Ki67 expression and MVD in the mouse model

**Fig. 4:**
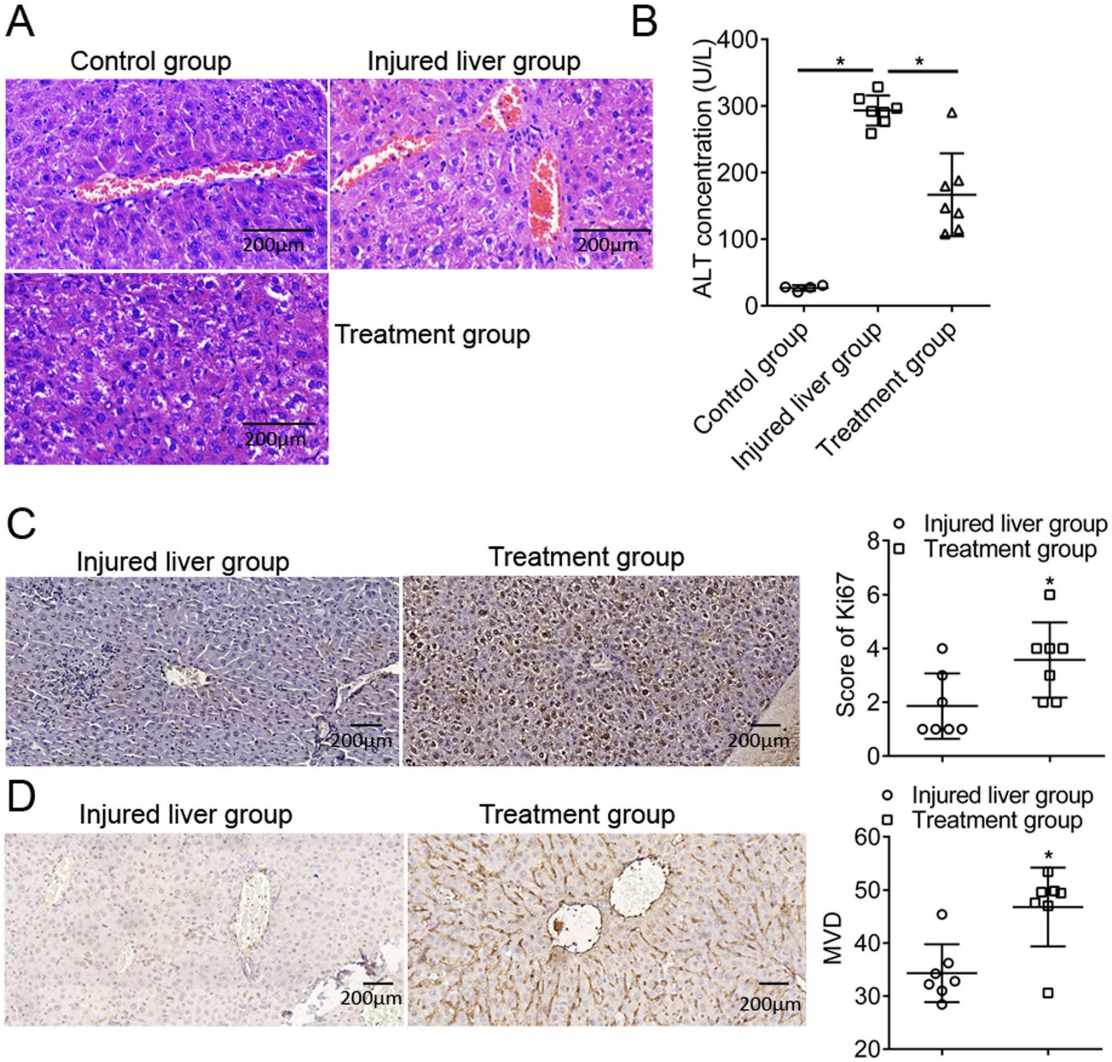
Granulocyte-colony stimulating factor (G-CSF) relieves liver injury and is positively correlated with the expression of Ki67 and microvessel density (MVD) in the mouse model. **A** Haematoxylin and eosin staining of liver tissue samples from mice in the control, injured liver, and treatment groups (× 200). **B** Alanine aminotransferase level in the serum of mice in the control, injured liver, and treatment groups. Immunohistochemical staining for Ki67 (**C**) and CD31 (**D**) in mouse liver tissue from the injured liver and treatment groups (× 200), and Scatter plot for Ki67 expression and MVD. *p < 0.05

After the peritoneal injection of D-GalN (1000 mg/kg), HE staining showed obvious liver injury (Fig. 4A), and mice with injured liver showed significantly higher serum ALT levels (293.24 ± 22.55 U/L; *p* < 0.001) than control mice (26.72 ± 4.23 U/L; Fig. 4B). These results confirmed the successful establishment of the injured liver mouse model. Furthermore, HE staining indicated less severe liver injury in G-CSF (250 µg/kg)-treated mice compared with injured and untreated mice (Fig. 4A). G-CSF-treated mice also showed significantly lower serum ALT levels (166.73 ± 62.12 IU/L; *p* = 0.003) than untreated mice with injured liver (293.24 ± 22.55; Fig. 4B). This further demonstrated the liver injury-repairing role of G-CSF. Moreover, the IHC staining score for Ki67 was significantly higher in the treatment group (4.04 ± 1.34; *p* = 0.002) than in the injured untreated liver group (2.5 ± 1.27; Fig. 4C). Additionally, MVD was significantly higher in the treatment group (47.78 ± 5.86; *p* < 0.001) than in the injured untreated liver group (35.06 ± 5.46; Fig. 4D). These results confirmed the existence of a positive correlation between G-CSF and viability and angiogenesis in the injured liver.

### G-CSF promoted the expression of Ki67 and VEGF-A via the AKT and ERK signalling pathways

**Fig. 5:**
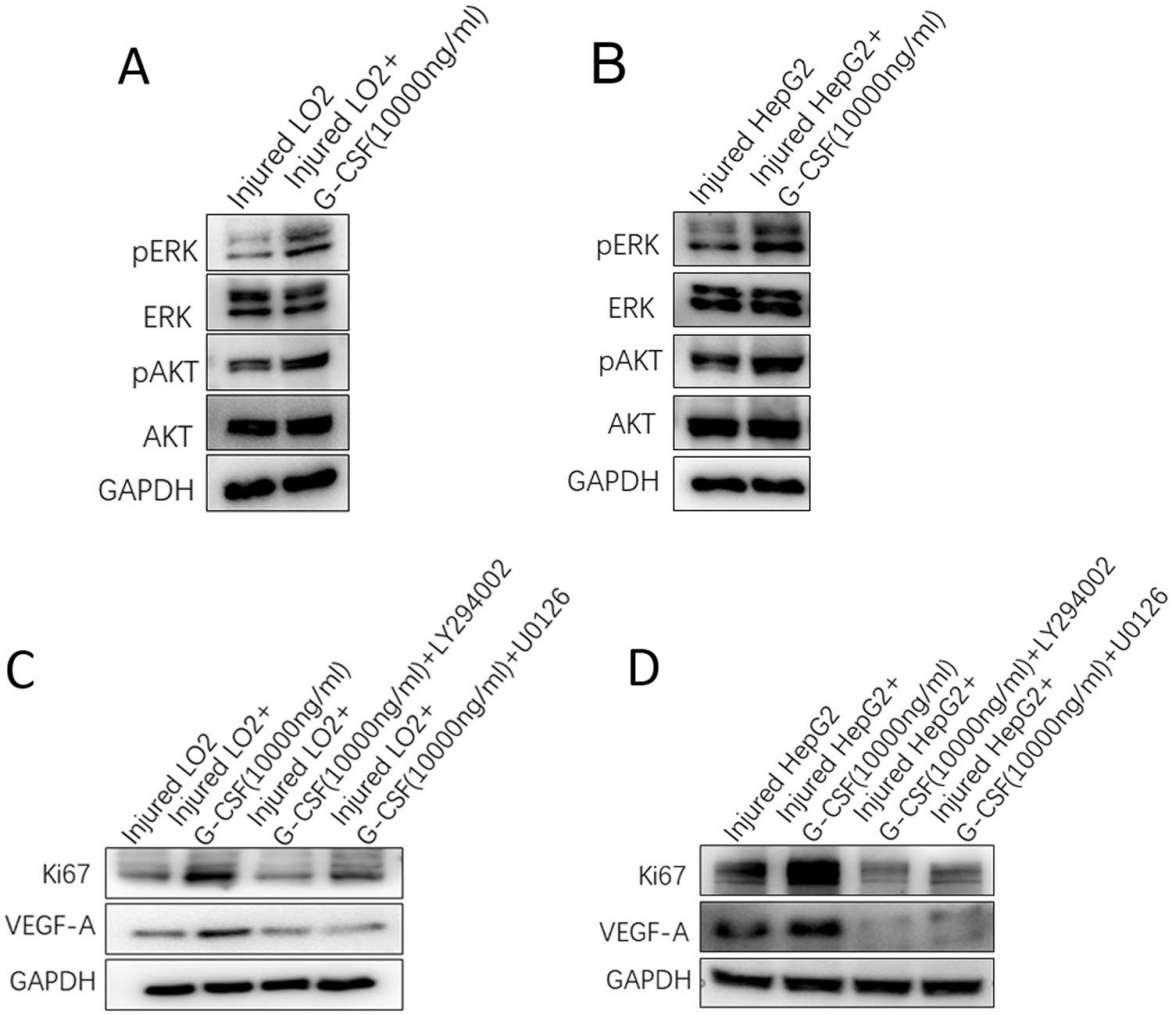
Granulocyte-colony stimulating factor (G-CSF) promotes the expression of Ki67 and VEGF-A via the AKT and ERK signalling pathways. **A** Changes in the phosphorylation of ERK and AKT in injured LO2 cells treated with G-CSF (10,000 ng/mL). **B** Changes in the phosphorylation of ERK and AKT in injured HepG2 cells treated with G-CSF (10,000 ng/mL). Expression of Ki67 and VEGF-A in injured LO2 + G-CSF (10,000 ng/mL) (**C**) and injured HepG2 + G-CSF (10,000 ng/mL) cells (**D**) treated with an ERK (U0126) or an AKT (LY294002) inhibitor

The levels of p-AKT and p-ERK in the injured LO2 and HepG2 cells increased significantly after treatment with 10,000 ng/mL G-CSF, whereas those of total AKT and ERK showed no significant changes (Fig. 5A, B). This implies that the AKT and ERK signalling pathways might play an important role in the observed effects of G-CSF. Additionally, pre-treatment of injured LO2 and HepG2 cells with the AKT inhibitor, LY294002, or the ERK inhibitor, U0126, significantly abrogated the G-CSF-induced increase in Ki67 and VEGF-A expression (Fig. 5C, D). These findings indicate that G-CSF can upregulate the expression of Ki67 and VEGF-A in injured liver cells via the AKT and ERK signalling pathways.

## Discussion

G-CSF, a 19.6-kDa glycoprotein, is a member of the colony-stimulating factor (CSF) family and is secreted by various cell types [15]. Its role in the treatment of neutropenia resulting from different causes and its neuroprotective, anti-apoptotic, and anti-inflammatory effects have been reported [16, 17]. However, in recent years, it has been reported that G-CSF is involved in the repair of LF in mouse models and human patients. In 2005, Yannaki et al. found that at an HSC-mobilization dose, G-CSF could ameliorate liver damage in an acute and chronic chemically injured mice model and could also significantly improve survival [18]. Furthermore, Garg et al. reported that the subcutaneous injection of G-CSF increases survival and decreases complications in patients with ACLF compared with placebo and standard medical therapy [19].

Presently, the effect of G-CSF on liver damage is attributed to its ability to stimulate HSC differentiation into liver cells [2, 11]. However, it is still not clear whether G-CSF can directly affect liver cells. To improve understanding in this regard, we used LO2 and HepG2 cells to establish an injured cell model. We observed that injured liver cells showed increased G-CSF and G-CSFR levels, which could be explained by the self-protection mechanism in the injured liver, implying that G-CSF plays a protective role in the injured liver. We also found that G-CSF increased the expression of Ki67 and the viability of the injured liver cells, highlighting the direct protective effects of G-CSF on injured liver cells. Furthermore, G-CSF repaired liver injury and upregulated Ki67 expression in the injured liver tissue. These findings provide sufficient evidence regarding the role of G-CSF in promoting liver viability by directly acting on liver cells.

Vasculature, an important part of the liver, is responsible for the delivery of nutrients and oxygen and the removal of toxic substances to ensure efficient liver functioning [20]. Endothelial integrity also plays a critical role in facilitating the functioning of liver vessels [21]. In patients with LF, liver sinusoidal endothelial cell damage and the disruption of microcirculation can aggravate liver injury, leading to a poor prognosis [22]. VEGF-A, a well-acknowledged proangiogenic factor, maintains vascular integrity by promoting the growth of endothelial cells [23]. Using a neurodegenerative model, Chen et al. found that G-CSF promoted the expression of VEGF-A in glial cells [24]. Whether G-CSF also promotes angiogenesis in the injured liver remained unknown. However, in this study, the in vitro findings demonstrated that G-CSF could promote the expression of VEGF-A in injured liver cells. Moreover, the supernatant from injured liver cells treated with G-CSF could enhance the proliferation and tubule formation ability of HUVECs. Additionally, in vivo, G-CSF repaired liver injury and increased the MVD in the injured liver tissue. These findings provide further evidence regarding the role of G-CSF in promoting angiogenesis through a direct effect on liver cells.

AKT and ERK are crucial signalling pathways for the maintenance of cellular functions, such as proliferation, angiogenesis, and migration under different conditions [25–28]. Walker et al. reported that the stimulation of endothelial insulin receptors promoted VEGF-A signalling and angiogenesis through ERK1/2 [29]. Moreover, Yang et al. observed that anlotinib inhibits the proliferation of colorectal cancer by blocking the AKT/ERK signalling pathway [30]. To further explore potential molecular mechanisms, we investigated the involvement of the AKT and ERK signalling pathways in the observed effects of G-CSF. G-CSF promoted the expression of Ki67 and VEGF-A in injured liver cells via the AKT and ERK signalling pathways, indicating their involvement in the molecular mechanism underlying the effect of G-CSF on liver cells. This is in accordance with the results of the previous studies [29, 30].

This study has some limitations. First, the repair of LF is complicated and can be affected by various factors, such as proliferation, angiogenesis, apoptosis inhibition, and autophagy. Although G-CSF promoted viability and angiogenesis, whether G-CSF also stimulates other mechanisms of repair needs further investigation. Second, considering the role of G-CSF in promoting the malignant phenotype of colon and gastric cancers [31] as well as its effects on liver cells observed in this study, it could possibly stimulate the malignant transformation of liver cells. Further studies are needed to provide clarifications in this regard.

## Conclusions

We demonstrated, for the first time, the effect of G-CSF in promoting the viability and angiogenesis of injured liver through direct effects on liver cells via the AKT and ERK signalling pathways (supplement Fig. 1). We elucidated the protective role of G-CSF in LF from a perspective that is different from those reported previously. Thus, this study improves understanding of the role of G-CSF in repairing liver damage and provides a solid theoretical basis for the clinical treatment of LF using G-CSF.

## Supplementary Information

Below is the link to the electronic supplementary material.

Supplementary file1 (TIF 1741 kb)—Proposed model for the promotion of injured liver cell viability and angiogenesis by granulocyte colony-stimulating factor (G-CSF). G-CSF stimulates the AKT and ERK signalling pathways, promoting Ki67 and VEGF-A expression in the injured liver cells. VEGF-A is secreted from injured liver cells and binds to VEGFR on HUVECs, promoting the proliferation of HUVECs and tubule formation, resulting in increased injured liver viability and angiogenesis.

Supplementary file2 (DOCX 13 kb)

Supplementary file3 (DOCX 13 kb)

## Data Availability

All data generated or analyzed during this study are included in this published article.
