# Supplementary figures and images for: G-CSF promotes the viability and angiogenesis of injured liver via direct effects on the liver cells
Source: Mol Biol Rep. 2022 Jul 4;49(9):8715–25. doi: 10.1007/s11033-022-07715-4 (PMC9463201; doi:10.1007/s11033-022-07715-4)

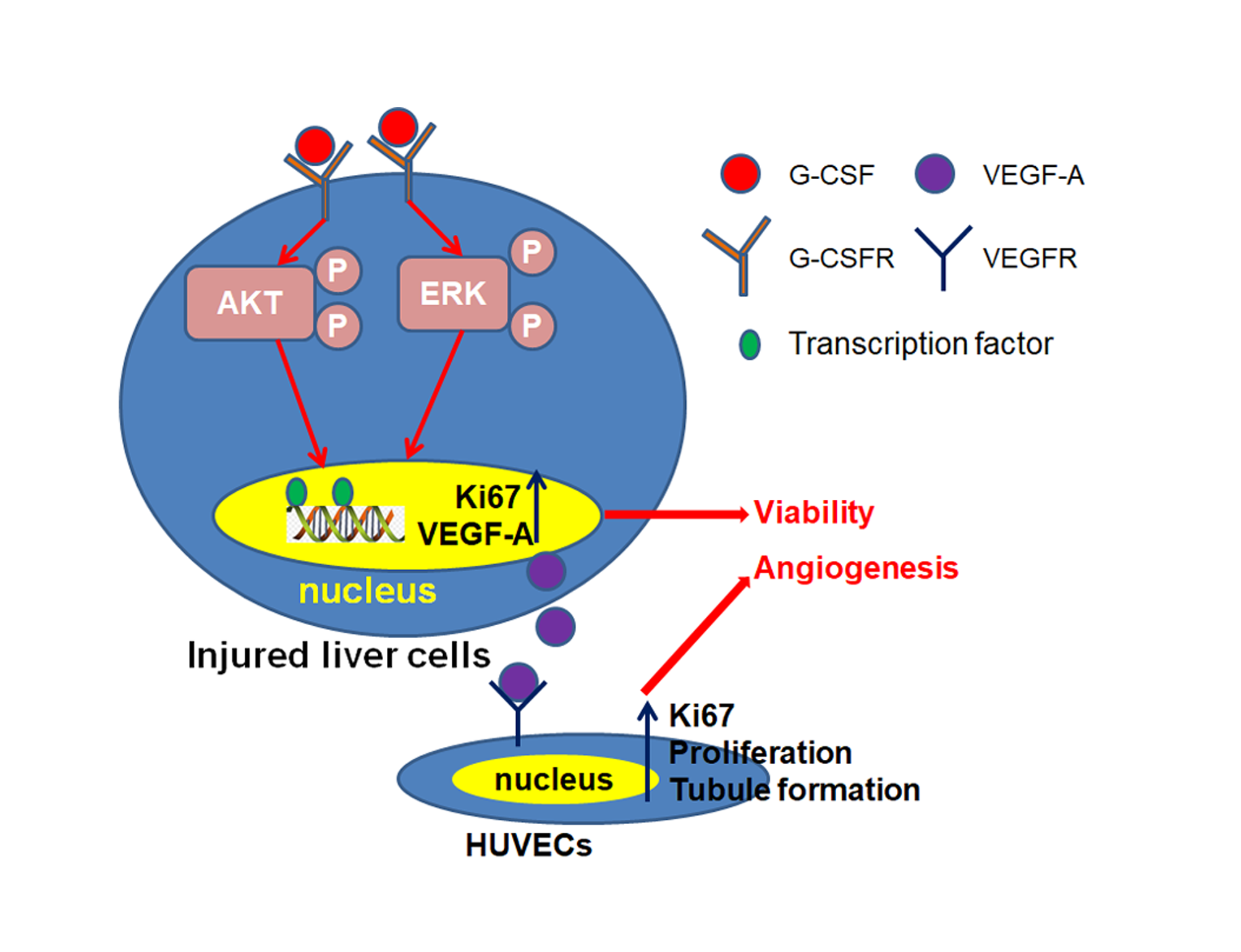

Supplement: Supplementary file 1 — Supplementary file1 (TIF 1741 kb)—Proposed model for the promotion of injured liver cell viability and angiogenesis by granulocyte colony-stimulating factor (G-CSF). G-CSF stimulates the AKT and ERK signalling pathways, promoting Ki67 and VEGF-A expression in the injured liver cells. VEGF-A is secreted from injured liver cells and binds to VEGFR on HUVECs, promoting the proliferation of HUVECs and tubule formation, resulting in increased injured liver viability and angiogenesis. [file 11033_2022_7715_MOESM1_ESM.tif]
