# Supplementary material for: G-CSF promotes the viability and angiogenesis of injured liver via direct effects on the liver cells
Source: Mol Biol Rep. 2022 Jul 4;49(9):8715–25. doi: 10.1007/s11033-022-07715-4 (PMC9463201; doi:10.1007/s11033-022-07715-4)
Supplement: Supplementary file 2 — Supplementary file2 (DOCX 13 kb) [file 11033_2022_7715_MOESM2_ESM.docx]

| **Name** | **Sequence** |
| --- | --- |
| G-CSFR | F 5’- CCTTGGAGGCCCTTCAGTAT-3’  R 5’- CCACCATGTGTCCAGTCTGA-3’ |
| Ki67 | F 5’-AGAAGAAGTGGTGCTTCGGAA-3’  R 5’- AGTTTGCGTGGCCTGTACTAA-3’ |
| VEGF-A | F 5’- AGGGCAGAATCATCACGAAGT-3’  R 5’- AGGGTCTCGATTGGATGGCA-3’ |
| GAPDH | F5’-CTGACTTCAACAGCGACACC-3’  R 5’-TGCTGTAGCCAAATTCGTTGT-3’ |

**Table 1 Primers used in qRT-PCR**
