# Supplementary material for: G-CSF promotes the viability and angiogenesis of injured liver via direct effects on the liver cells
Source: Mol Biol Rep. 2022 Jul 4;49(9):8715–25. doi: 10.1007/s11033-022-07715-4 (PMC9463201; doi:10.1007/s11033-022-07715-4)
Supplement: Supplementary file 3 — Supplementary file3 (DOCX 13 kb) [file 11033_2022_7715_MOESM3_ESM.docx]

| **Name** | **Dilution Ratio** | **Company** |
| --- | --- | --- |
| G-CSFR | 1:500 | ab126167, abcam, Cambridge, MA, U.S. |
| Ki67 | 1:1000 | ab16667, abcam, Cambridge, MA, U.S. |
| VEGF-A | 1:500 | ab183100, abcam, Cambridge, MA, U.S. |
| phospho-ERK1/2 | 1:1000 | 8544, CST, Cell Signaling Technology, Danvers, MA, USA |
| ERK1/2 | 1:1000 | 4348, CST, Cell Signaling Technology, Danvers, MA, USA |
| phospho-Akt | 1:1000 | 13038, CST, Cell Signaling Technology, Danvers, MA, USA |
| Akt | 1:1000 | 4691, CST, Cell Signaling Technology, Danvers, MA, USA |
| GAPDH | 1:10000 | 60004-1 , Proteintech Group, Inc., Wuhan, China |

**Table 2 Primary antibodies used in Western blot**
